# Physicochemical, Mechanical, and Antimicrobial Properties of Novel Dental Polymers Containing Quaternary Ammonium and Trimethoxysilyl Functionalities

**DOI:** 10.3390/jfb11010001

**Published:** 2019-12-18

**Authors:** Diane R. Bienek, Anthony A. Giuseppetti, Stanislav A. Frukhtbeyn, Rochelle D. Hiers, Fernando L. Esteban Florez, Sharukh S. Khajotia, Drago Skrtic

**Affiliations:** 1 ADA Foundation, Research Division, Frederick, MD 21704, USA; giuseppettia@ada.org (A.A.G.); frukhtbeyns@ada.org (S.A.F.); dskrtic@verizon.net (D.S.); 2 College of Dentistry, University of Oklahoma Health Sciences Center, Oklahoma City, OK 73117, USA; Shelley-Hiers@ouhsc.edu (R.D.H.); fernando-esteban-florez@ouhsc.edu (F.L.E.F.); Sharukh-Khajotia@ouhsc.edu (S.S.K.)

**Keywords:** antimicrobial effect, biofilms, cytotoxicity, dental resins, physicochemical properties, mechanical properties, quaternary ammonium methacrylates

## Abstract

The aims of this study were to evaluate the physicochemical and mechanical properties, antimicrobial (AM) functionality, and cytotoxic potential of novel dental polymers containing quaternary ammonium and trimethoxysilyl functionalities (e.g., N-(2-(methacryloyloxy)ethyl)-*N*,*N*-dimethyl-3-(trimethoxysilyl)propan-1-aminium iodide (AM_sil1_) and N-(2-(methacryloyloxy)ethyl)-*N*,*N*-dimethyl-11-(trimethoxysilyl)undecan-1-aminium bromide (AM_sil2_)). AM_sil1_ or AM_sil2_ were incorporated into light-cured (camphorquinone + ethyl-4-*N*,*N*-dimethylamino benzoate) urethane dimethacrylate (UDMA)/polyethylene glycol-extended UDMA/ethyl 2-(hydroxymethyl)acrylate (EHMA) resins (hereafter, UPE resin) at 10 or 20 mass %. Cytotoxic potential was assessed by measuring viability and metabolic activity of immortalized mouse connective tissue and human gingival fibroblasts in direct contact with monomers. AM_sil_–UPE resins were evaluated for wettability by contact angle measurements and degree of vinyl conversion (DVC) by near infra-red spectroscopy analyses. Mechanical property evaluations entailed flexural strength (FS) and elastic modulus (E) testing of copolymer specimens. The AM properties were assessed using *Streptococcus mutans* (planktonic and biofilm forms) and *Porphyromonas gingivalis* biofilm. Neither AM_sil_ exhibited significant toxicity in direct contact with cells at biologically relevant concentrations. Addition of AM_sil_s made the UPE resin more hydrophilic. DVC values for the AM_sil_–UPE copolymers were 2–31% lower than that attained in the UPE resin control. The mechanical properties (FS and E) of AM_sil_–UPE specimens were reduced (11–57%) compared to the control. Compared to UPE resin, AM_sil1_–UPE and AM_sil2_–UPE (10% mass) copolymers reduced *S. mutans* biofilm 4.7- and 1.7-fold, respectively (*p* ≤ 0.005). Although not statistically different, *P. gingivalis* biofilm biomass on AM_sil1_–UPE and AM AM_sil2_–UPE copolymer disks were lower (71% and 85%, respectively) than that observed with a commercial AM dental material. In conclusion, the AM function of new monomers is not inundated by their toxicity towards cells. Despite the reduction in mechanical properties of the AM_sil_–UPE copolymers, AM_sil2_ is a good candidate for incorporation into multifunctional composites due to the favorable overall hydrophilicity of the resins and the satisfactory DVC values attained upon light polymerization of AM_sil_-containing UDMA/PEG-U/EHMA copolymers.

## 1. Introduction

As people live longer and retain more of their own teeth, the incidence of dental caries, especially root caries, increases. Currently, the prevalence of root caries in older adults ranges from 29% to 89% [1]. The expected aging of the population will further increase root caries occurrences. Therefore, implementing more effective prevention strategies and/or developing new treatments for root caries is prudent. Often, compromised integrity of the conventional restorative/tooth interface [2,3] ultimately results in bacterial microleakage and secondary caries. Class V restoratives may release fluoride ions which, at adequate concentrations, protect teeth from demineralization and possibly contribute to regeneration of mineral lost to caries. Fluoride release, however, does not provide an effective antimicrobial (AM) protection, although fluoride can have some AM properties [4,5]. The majority of the contemporary dental restoratives do not possess substantial AM properties [6] verifiable in clinical trials [7]. To improve the longevity of repair, the restorative material should be AM. Adding AM function to dental materials typically focuses on release/slow release of various low molecular weight AM agents [5,8,9,10,11,12,13,14,15]. However, mechanism(s) of their actions are elusive, and there are concerns about their toxicity to human cells, the development of tolerances (in the case of antibiotics), and long-term efficacies. Moreover, the release of these agents can compromise mechanical performance of the restoratives and, if the dose or release kinetics are not properly controlled, can induce toxicity to the surrounding tissues [16].

Antimicrobial polymeric materials with quaternary ammonium (QA) salts have been widely applied to a variety of antimicrobial-relevant areas (reviewed by [17,18]). QA methacrylates are known for their AM action against both Gram-positive and Gram-negative bacteria. Studies have indicated that QA compounds destroy bacterial cell membrane integrity and eventually lead to cell death [19,20,21]. The proposed mechanism of action is the electrostatic interaction between positively charged molecules and negatively charged microbial cell membranes. So far, QA methacrylates have not been successfully incorporated into dental restorative(s) to yield a sustained AM function [22]. Historically, the most attention has been given to methacryloyloxydodecyl pyrimidinium bromide (MDPB) and its acrylamide copolymer [6,23]. MDBP has been commercialized and suggested to be potentially applicable to various restoratives. However, due to their poor color stability, MDBP-based materials can only be used for aesthetically inferior restorations. To widen the utility of QAs in restorative dentistry, various QAs have been formulated into bonding agents and dental resin composites [20,24,25,26]. Successful incorporation of these new AM QAs into polymeric phases of composite materials would be a major step in creating new Class V restoratives that are clinically effective against secondary caries. We are advancing the development of Class V restorative materials by introducing bioactive amorphous calcium phosphate (ACP) filler into polymer-based restorative in parallel to the AM monomer, thus creating a multifunctional AM and remineralizing materials (hereafter AMRE). ACP has been indicated as a precursor to hydroxyapatite formation both in vitro and in vivo [27,28,29,30,31,32,33]. ACP also exhibited favorable in vivo osteoconductivity compared to hydroxyapatite [34]. Based on our group’s knowledge of ACP chemistry and our understanding of structure/composition/property relationships existing in ACP polymeric systems [35,36,37,38,39], we have undertaken a task to formulate AMRE polymeric composites that maintain a desired state of supersaturation with respect to hydroxyapatite and efficiently restore mineral-depleted tooth structures while providing sustained AM protection. Prior to formulating AMRE composites, it is essential to evaluate the AM-containing resins (no ACP filler) to establish the effect of AM monomers on basic biological, physicochemical, and mechanical properties of copolymers.

**Figure 1:**
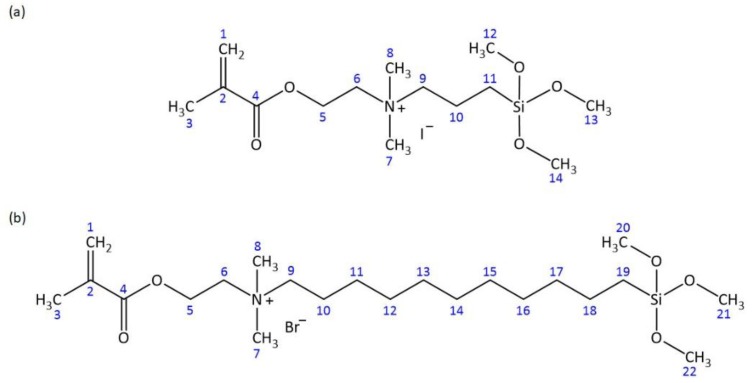
Skeletal structural formulas of AM_sil1_ (**a**) and AM_sil2_ (**b**) monomers.

AM_sil_ syntheses and subsequent validation protocols of novel dental monomers containing QA and trimethoxysilyl functionalities are described in detail by Okeke et al. (2019) [40]. The motivation for synthesizing AM_sil1_ and AM_sil2_ was to develop coupling agents capable of conferring the AM properties and coupling with both ACP phase and polymer phase of Class V resin-based composites. This study reports on the incorporation of these two polymerizable QA monomers with different alkyl chain lengths (e.g., N-(2-(methacryloyloxy)ethyl)-*N*,*N*-dimethyl-3-(trimethoxysilyl)propan-1-aminium iodide (AM_sil1_) and N-(2-(methacryloyloxy)ethyl)-*N*,*N*-dimethyl-11-(trimethoxysilyl)undecan-1-aminium bromide (AM_sil2_)) (Figure 1) into UDMA/poly(ethylene glycol)-extended UDMA (PEG-U)/ethyl 2-(hydroxymethyl) acrylate (EHMA) resins (hereafter UPE resin) and the biological, physicochemical, and mechanical screening of AM–UPE copolymers. Working hypotheses were that AM_sil_ monomers will show minimal or no toxicity towards immortalized mouse subcutaneous connective tissue fibroblasts (CCL1) or human gingival fibroblasts (HGF) and that AM_sil_–UPE copolymers will have similar physicochemical and mechanical properties compared to the parent UPE copolymers. AM_sil1_– and AM_sil2_–UPE copolymers were assessed for their AM activity against *Streptococcus mutans* and *Porphyromonas gingivalis,* which are model microorganisms for dental caries [41,42] and periodontal disease [43,44], respectively.

## 2. Results

### 2.1. Structural Verification of AM_sil_s

The AM_sil_ structures were verified by ^1^H and ^13^C NMR (Table 1 and Table 2). In brief, both ^1^H and ^13^C NMR spectra of AM_sil_s confirmed the successful quaternization of 2-(dimethylamino)ethyl methacrylate (DMAEMA) precursor. By performing the syntheses in chloroform, methoxy groups of (3-iodopropyl)trimethoxy silane (IPTMS) and (11-bromoundecyl)trimethoxy silane (BrUDTMS) were protected from hydrolysis to which they are prone in aqueous environment.

### 2.2. Biocompatibility Testing

#### 2.2.1. AM_sil1_

**Figure 2:**
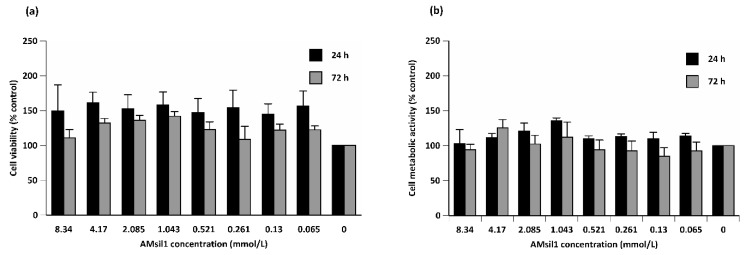
Percent control value of viability (**a**) and metabolic activity (**b**) of CCL1 cells exposed to 2-fold serial dilutions of AM_sil1_ (≤8.34 mmol/L) for 24 or 72 h. Data represent mean ± standard error for five independent replicates tested in triplicate.

AM_sil1_ concentration did not exert a statistically significant effect on the viability of CCL1 cells (Figure 2). However, the exposure time reduced the number (1.2-fold difference of mean, *p* ≤ 0.002) of live cells. Similarly, AM_sil1_ concentration did not affect the metabolic activity of CCL1 cells. Time of exposure had a modest effect (*p* ≤ 0.02) on CCL1 metabolic activity, although no significant paired comparisons (within each concentration) were observed.

**Figure 3:**
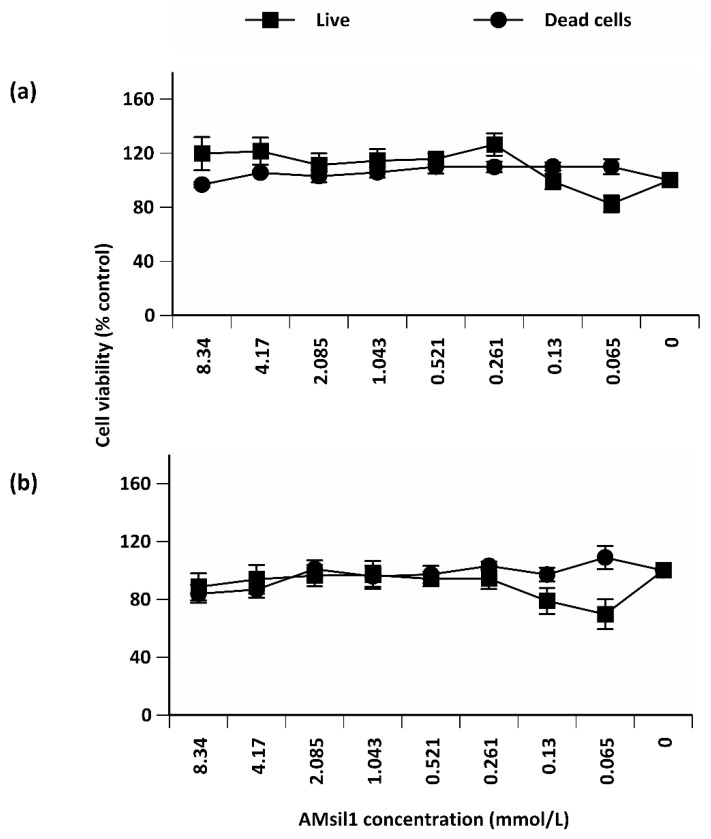
Percent control value of viability of human gingival fibroblast (HGF) cells exposed to 2-fold serial dilutions of AM_sil1_ (≤8.34 mmol/L) for (**a**) 24 h or (**b**) 72 h. Data represent mean ± standard error for five independent replicates tested in triplicate.

Independent of time, AM_sil1_ concentration exerted a main effect (*p* ≤ 0.001) on the number of viable HGFs (Figure 3). Paired comparisons indicated that at lower AM_sil1_ concentrations (≤0.13 mmol/L), the number of live HGFs decreased. Independent of AM_sil1_ concentration, exposure time reduced (19.73 difference of means, *p* ≤ 0.001) HGF viability. Monomer concentration or time of exposure did not statistically affect the metabolic activity of HGF cells (data not shown).

#### 2.2.2. AM_sil2_

**Figure 4:**
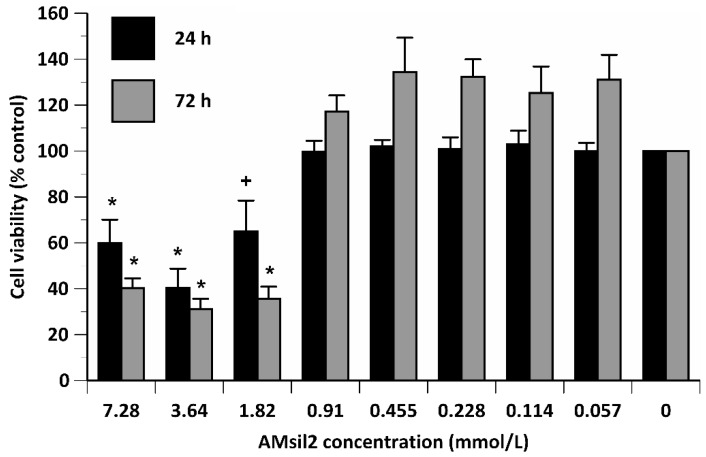
Percent control value of viability of CCL1 cells exposed to 2-fold serial dilutions of AM_sil2_ (≤7.28 mmol/L) for 24 or 72 h. Data represent mean ± SEM for five independent replicates tested in triplicate. * indicates *p* ≤ 0.05 when compared to concentrations ≤ 0.91 mmol/L within the same time period. + indicates *p* ≤ 0.05 when compared to 0.455, 0.228, or 0.114 mmol/L concentrations within same time period.

Regardless of time, AM_sil2_ concentration exerted a main effect (*p* ≤ 0.001) on the number of viable CCL1s (Figure 4). Paired comparisons indicated that at 24 h exposure, CCL1 cells exposed to ≥3.64 mmol/L were lower (*p* ≤ 0.05) than the number of live cells exposed to all lower concentrations. At 72 h exposure, the percentage of live CCL1 cells exposed to ≥1.82 mmol/L AM_sil2_ was at least 3- to 4-fold lower (*p* ≤ 0.001) compared to all concentrations ≤ 0.91 mmol/L.

Like cell viability, AM_sil2_ concentration exerted an effect (*p* ≤ 0.001) on CCL1 metabolic activity. Viability and metabolic activity of CCL1 cells exposed to AM_sil2_ showed a strong positive linear correlation at 24 h (R^2^ = 0.91, *p* ≤ 0.0005) and 72 h (R^2^ = 0.93, *p* ≤ 0.0005) (data not shown).

**Figure 5:**
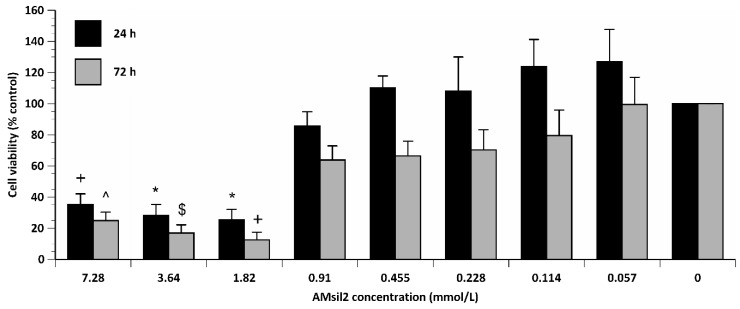
Percent control value of viability of HGF cells exposed to 2-fold serial dilutions of AM_sil2_ (≤7.28 mmol/L) for 24 h or 72 h. Data represent mean ± SEM for five independent replicates tested in triplicate. + indicates *p* ≤ 0.05 when compared to concentrations ≤ 0.455 mmol/L within same time period. * indicates *p* ≤ 0.05 when compared to concentrations ≤ 0.91 mmol/L within same time period. ^ indicates *p* ≤ 0.05 when compared to concentrations ≤ 0.114 mmol/L within same time period. $ indicates *p* ≤ 0.05 when compared to concentrations ≤ 0.228 mmol/L within same time period.

AM_sil2_ exhibited a concentration effect (*p* ≤ 0.001) on HGF cell viability (Figure 5). Paired comparisons indicated that exposure to ≥1.82 mmol/L AM_sil2_ reduced the number of live HGFs by more than 3-fold (*p* ≤ 0.05) compared to the control group. When considering the effect of time regardless of AM_sil2_ concentration, the number of live cells was consistently lower (~23% difference of means). Like viability, AM_sil2_ concentration exhibited an effect (*p* ≤ 0.001) on HGF metabolic activity. At 24 and 72 h, viability and metabolic activity of HGF cells exposed to AM_sil2_ showed a positive linear correlation (R^2^ = 0.94; (*p* ≤ 0.0005) and R^2^ = 0.77; (*p* ≤ 0.001), respectively) (data not shown).

For both AM_sil_s, control wells (with or without cells) in which the tetrazolium salt reagent was omitted resulted in negligible optical density values. Positive control wells containing unexposed cells (i.e., no AM_sil_s) that were given an equal volume of culture medium were not significantly different from cells that were previously treated with the viability stain (data not shown).

### 2.3. Hydrophobicity/Hydrophilicity of the Resins

**Figure 6:**
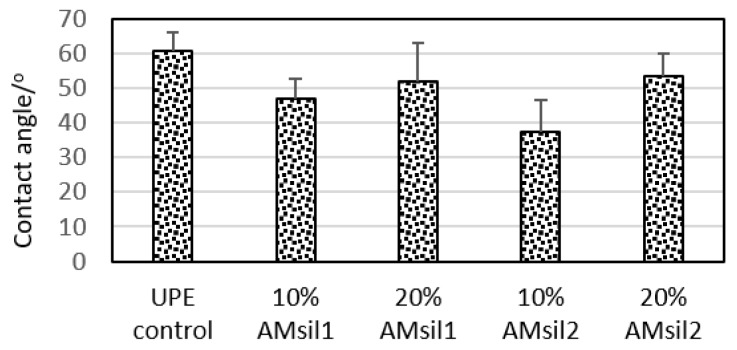
The contact angle (CA) values of AM_sil_–UPE and UPE control indicative of the changes in resin’s overall hydrophilicity/hydrophobicity upon introduction of AM_sil_ monomers at 10 and 20 mass % relative to UPE. Shown are mean values + standard deviation of four repetitive measurements in each experimental group.

Copolymers comprised of UPE resin with added AM_sil_s generally exhibited lower contact angles (CAs) (Figure 6), suggesting change in their hydrophilic/hydrophobic balance toward more hydrophilic surfaces. At 10 mass % monomer in the resin, CAs of both AM_sil1_–UPE and AM_sil2_–UPE copolymers (46.9 ± 5.9° and 37.4 ± 9.2°, respectively) were significantly lower (23% and 38% reduction, respectively; *p* ≤ 0.01) than the CA of the UPE control 60.8 ± 5.1°. The apparent increase in the CA in going from 10% to 20% AM_sil_ in the resin was significant only for AM_sil2_ (*p* ≤ 0.035). The overall order of the decreasing relative hydrophilicity (evidenced by the increasing CA values) of the examined UPE-based copolymers was as follows: (10% AM_sil2_–UPE ≥ 10% AM_sil1_–UPE) > (20% AM_sil2_–UPE = 20% AM_sil1_–UPE) > UPE control.

### 2.4. Effect of AM_sil_s on Degree of Vinyl Conversion (DVC)

**Figure 7:**
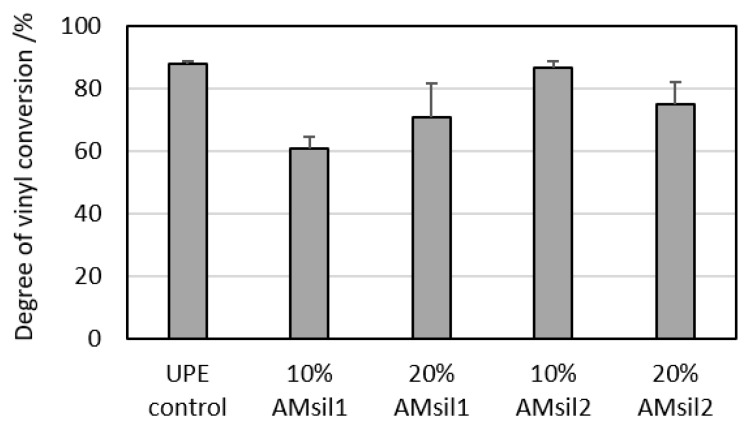
The values for degree of vinyl conversion (DVC) attained 24 h post-cure in AM_sil_–UPE copolymers compared to no-AM UPE control. Shown are mean values + standard deviation of three repetitive measurements.

Introduction of 10% and 20% AM_sil1_ into UPE reduced (*p* ≤ 0.05) the mean vinyl moiety conversion upon photopolymerization by 31% and 20%, respectively (Figure 7). No significant effect was observed with the increasing levels of AM_sil1_ in the resin. Although reduced, the DVC observed amongst the AM_sil2_ groups was not statistically different from one another or the UPE control group.

### 2.5. Mechanical Properties of AM_sil_–UPE Copolymers

**Figure 8:**
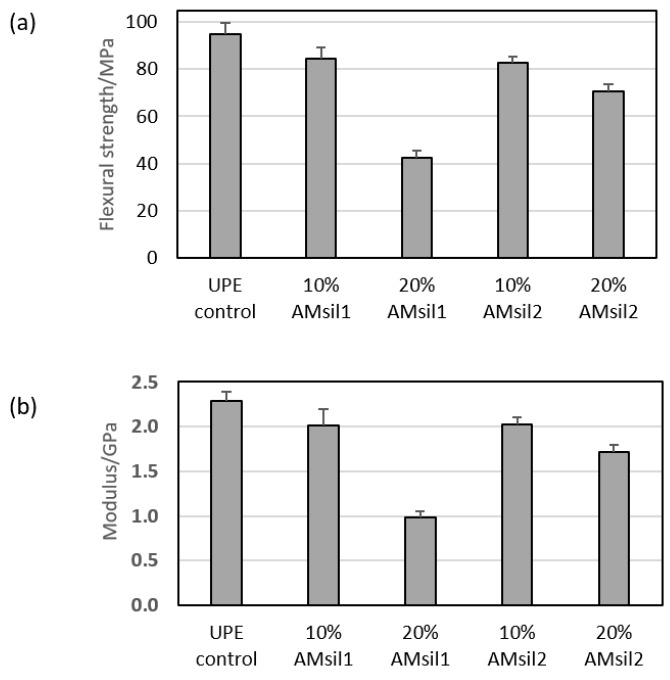
(**a**) Flexural strength and (**b**) tensile elasticity of AM_sil_–UPE copolymers in comparison with the UPE control. Indicated are mean values + standard deviation of three specimens.

The FS and E of AM_sil_–UPE copolymers were, generally, diminished compared to the UPE resin control (Figure 8). The extent of reduction in FS and E varied with the type and the concentration of AM_sil_. In all AM_sil_–UPE formulations, the FS values were significantly (*p* ≤ 0.05) lower than the UPE control counterparts. In both 10 mass % AM_sil_ formulations, the E was reduced, although not statistically significant. At 20 mass %, the E of both AM_sil_ formulations were notably lower (*p* ≤ 0.0008) than the UPE resin control. Both FS and E reductions ranged from moderate (11–13%) for 10 mass % AM_sil_ formulations to substantial (25–57%) for 20 mass % AM_sil_ formulations.

### 2.6. Bacterial Testing

**Figure 9:**
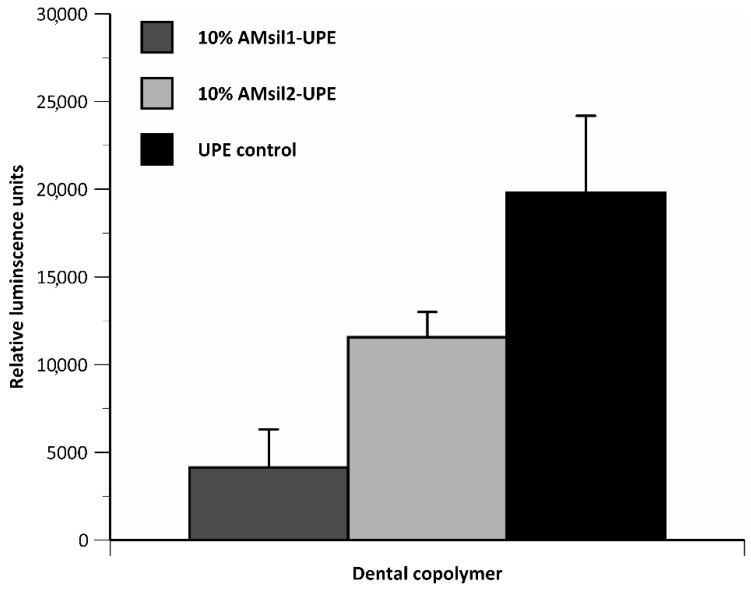
*Streptococcus mutans* biofilm growth inhibition by the experimental AM_sil_s–UPE (10 mass %) copolymers compared to UPE control resin. Bar height indicates mean + standard deviation of 5 specimens/group.

For planktonic bacterial testing, the number of *S. mutans* colony-forming units/mL observed amongst the AM_sil_ groups were not statistically different from one another or the control groups (UPE only and commercial control). However, compared to UPE resin, AM_sil1_–UPE and AM_sil2_–UPE (10% mass) copolymers reduced the colonization of *S. mutans* biofilm 4.7- and 1.7-fold, respectively (*p* ≤ 0.002) (Figure 9). *S. mutans* biofilms exposed to AM_sil1_–UPE were at least 2.8-fold lower (*p* ≤ 0.005) than that observed with AM_sil2_–UPE.

**Figure 10:**
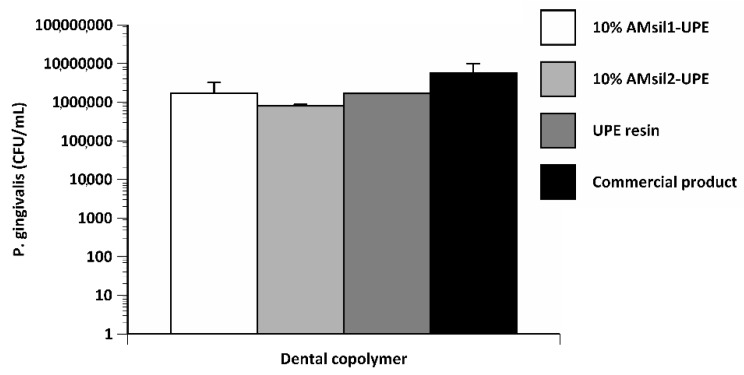
*Porphyromonas gingivalis* biofilm growth inhibition by the experimental AM_sil_s–UPE (10 mass %) copolymers compared to UPE control resin. Bar height indicates mean + standard deviation of 5 specimens/group.

*P. gingivalis* biofilm biomass on copolymer disks exposed to AM_sil1_–UPE and AM_sil2_–UPE were lower (71% and 85%, respectively) than that observed with the commercial control, albeit not statistically different (*p* ≤ 0.07) (Figure 10).

## 3. Discussion

We considered cytotoxicity of any AM monomer to be a major determinant of whether the materials that incorporate the monomer in their resin phase are worthy of further study. We believe that direct contact cellular testing of the new agent must be done at biologically relevant eluent concentrations. In this study, we employed conditions that reflect the accelerated leachability of UPH resins and included AM_sil_ concentrations that significantly exceed the upper thresholds established experimentally in the previous work [45]. The direct toxicity of AM_sil_s towards CCL1 cells and/or HGFs was demonstrated to be marginal or undetectable, except at the higher concentrations of monomers tested. These high AM_sil_ levels correspond to unrealistically high levels of the unreacted monomers and are highly unlikely to ever be registered clinically. Our cytotoxicity results support the basic hypothesis of the study and suggest that, from a biotoxicity viewpoint, AM_sil_s can be safely utilized in design of AM new materials. Leachability studies of AM–UPE formulations employing high-performance liquid chromatography are currently underway in our laboratory, and are expected to confirm the conclusions derived from the tests based on the accelerated UPH leachability study.

Upon introduction of AM_sil_ into the UPE resin, a shift towards lower CA values, consistent with the moderate increase in the overall hydrophilicity, was seen in all AM_sil_–UPE formulations. The detected range of CAs in AM_sil_–UPE resins (37.4–53.3°) correlates very well with the range of CAs typical for the commercial resin composites (37.4–53.3°) [46]. The lowest CAs detected in 10% AM_sil2_–UPE formulation make this resin a good candidate for the incorporation of the ACP filler in future design of AMRE composites. ACP-filled composite requires sufficient water absorption to initiate water-catalyzed transformation of ACP during which the remineralizing calcium and phosphate ions are released, by diffusion, into surrounding mineral-deficient tooth structures. There, they regenerate these mineral-depleted structures via redeposition of hydroxyapatite [47]. Taken together, the enhanced wettability should ease a diffusion of water into AMRE composite and result in the subsequent release of calcium and phosphate ions from the composite needed for demineralization prevention and/or active remineralization at the restoration site.

The range of DVC values attained in AM_sil_–UPE copolymers (60.7–86.7%), dependent on both the monomer type and its quantity in the resin, were higher or equal to the DVC reported for 2,2-bis[p-(2-hydroxy-3-methacryloxypropoxy)phenyl]propane (bis-GMA)-based resins/composites with incorporated QA ionic dimethacrylate (67.9–70.7%) [25]. AM_sil2_ copolymers reached significantly higher DVC values (75.2–86.7%) compared to their AM_sil1_ counterparts (60.7–70.9%). In AM_sil2_–UPE formulations, the inclusion of AM monomer apparently did not affect the high levels of DVC typically seen in UDMA-based resins [35,36]. This phenomenon has been attributed to the chain transfer reactions caused by UDMA’s –NH– groups, resulting in increased mobility of the resin network’s radical sites [48]. The DVCs attained in AM_sil2_–UPE copolymers suggest limited mobility of cross-linked polymer matrix, thus reducing the likelihood of unreacted monomers leaching out to a minimum. The observed DVC decrease in AM_sil1_–UPE formulations compared to those of AM_sil2_–UPE is yet to be explained.

The results of FS and E tests indicated a reduction of the copolymers’ mechanical properties in going from the UPE control to 10% AM_sil_–UPE to 20% AM_sil_–UPE. The reduction was far more pronounced in AM_sil1_ series (50%) compared to the AM_sil2_ series (15%). This overall reduction in mechanical properties, particularly in AM_sil2_–UPE copolymers, should not disqualify this monomer from further exploration, as AM agent in multifunctional AMRE composites. To compensate for the reduction in the mechanical properties, incorporation into composites of the reinforcing fillers in addition to ACP should be considered.

AM_sil_s integrated into UPE resin were effective in reducing (*p* ≤ 0.002) *S. mutans* biofilms. Compared to the commercial control, *P. gingivalis* biofilm biomass was notably lower (71–85%) on AM_sil_s–UPE copolymer disks, however, not statistically different. As Gram-positive bacteria have peptidoglycan with long anionic polymers, called teichoic acids [49] (i.e., yielding a higher cell surface net negative charge than Gram-negative organisms), one could anticipate *S. mutans* to be more susceptible to AM_sil_s. Notwithstanding, quaternary ammonium compound AM functionality can also be affected by the type of counter-ion [50], pendant active groups [51], molecular weight, and length of the alkyl chains [52].

AM_sil_s show promise, as *S. mutans* planktonic and biofilm forms were reduced. Nonetheless, this reduction was only significant (*p* ≤ 0.002) for biofilms. When comparing planktonic and biofilm responses, similar trends were observed with an endodontic sealer. For example, bacteria tested with a resin-based root canal sealer did not statistically reduce the planktonic forms, while notably decreasing bacteria in monospecies biofilms [53]. These authors attributed this to the release of substances during the setting process. This attribute is unlikely applicable to our material, as DVC was high and aqueous extraction was conducted for 72 h. In another report, *Staphylococcus aureus* biofilms were demonstrated to be more susceptible to killing than the planktonic form of the same strain [54].

Although AM functionality is observed, we believe that the full potential of the AM_sil_ monomers has not yet been realized. As reported for other quaternary ammonium compounds [47], the current AM_sil_–UPE copolymer formulations are likely to have N^+^ charges randomly distributed throughout the material. As the mechanism of AM action is contingent on contact, it would be advantageous to develop fabrication methods that would favor charge density at the materials surface. Further, others have demonstrated that proteins can diminish the AM capability of quaternary ammonium methacrylates (reviewed by [6]). Currently, there is insufficient information concerning the interaction of proteins with quaternary ammonium methacrylates. Elucidation of the protein–material interactions would yield valuable information to develop strategies to maximize AM efficacy of materials with a charge-based AM mechanism of action.

In conclusion, our novel AM dental monomers (AM_sil1_ and AM_sil2_) exhibited minimal or no toxicity upon direct contact with biologically relevant concentrations, while reducing *S. mutans* and *P. gingivalis* biofilm forms. AM_sil_s made the UDMA/PEG-U/EHMA resin more hydrophilic. This would be an advantageous feature in AMRE composites that require water to induce their remineralizing effects. At 10 mass % level of AM_sil_ monomer, DVC of the ensuing AM_sil_–UPE copolymers was only marginally lower than in UPE control and still exceeded DVCs typically seen in the commercial composites based on bis-GMA/triethyleneglycol dimethacryalate (TEGDMA) resins. The mechanical properties of AM_sil_–UPE copolymers were reduced (11–57%) compared with the UPE control. The extent of reduction depended on both the type and the concentration of AM_sil_ monomer in the resin. This finding should not disqualify the AM_sil_–UPE resins from use in AMRE composites intended for Class V restorations where the mechanical stability is not a critical factor.

## 4. Materials and Methods

### 4.1. Monomer Synthesis

The synthesis and validation protocols for the AM_sil_s are described in detail by Okeke et al. (2019) [40]. In brief, AM_sil1_ and AM_sil2_ were synthesized at 50–55 °C by reacting equimolar amounts of tertiary amine, DMAEMA, with IPTMS and BrUDTMS, respectively, in the presence of chloroform and butylated hydroxytoluene. DMAEMA, IPTMS, and butylated hydroxytoluene were purchased from Sigma, St. Louis, MO, USA. BrUDTMS was purchased from Gelest Inc., Morrisville, PA, USA. Reactants and solvents (chloroform, diethyl ether, hexane; Sigma, St. Louis, MO, USA) used during synthesis and the subsequent purification were used as received, without further purification. The reaction yields were 94.8% and 36.0% for AM_sil1_ and AM_sil2_, respectively. Due to the generally hygroscopic nature of QA monomers, the AM_sil_s were stored under vacuum (25 mm Hg) before being used for resin formulation and/or copolymer disk specimen preparation.

### 4.2. Structural Verification

Purified monomers were characterized by ^1^H and ^13^C NMR spectroscopy as described [40]. Briefly, spectra were obtained using a Bruker Advance II (600 MHz) spectrometer equipped with a Broadband Observe room temperature probe (Bruker, Corp., Billerica, MA, USA). Monomers were dissolved in deuterated dimethyl sulfoxide containing tetramethylsilane.

### 4.3. Experimental Resin Formulation

UPE resin was formulated from the commercially available monomers UDMA, PEG-U, and EHMA at 2.8:1.0:1.7 mass ratio (corresponds to the average mass ratio of the ternary UDMA-based formulations explored by our group so far). A conventional visible light initiator system comprised of camphorquinone and ethyl-4-*N*,*N*-dimethylamino benzoate (4EDMAB) was introduced to the resin at concentrations of 0.2 mass % camphorquinone and 0.8 mass % 4EDMAB. AM_sil_s were blended into UPE resin to yield (AM_sil1_ or AM_sil2_)–UPE resin with 10 or 20 mass % of AM component. Addition of AM_sil1_ or AM_sil2_ to the light-activated UPE resin took place in the absence of blue light. The rationale for the chosen levels of AM monomers is based on the previously reported AM activities of similar QA methacrylates in camphorquinone /4EDMAB-activated bis-GMA/TEGDMA resins [25]. Once all components were introduced, the mixture was stirred magnetically (38 rad/s) at 22 °C until a uniform consistency was achieved. CLEARFIL SE Protect BOND (Kurary America, Inc., New York, NY, USA) was used as a comparative commercial AM material. This resin was prepared using equal quantities of primer (containing MDPB) and bonding (containing bis-GMA-HEMA) agent.

### 4.4. Biocompatibility Tests

Direct contact cytotoxicity of AM_sil_s was determined following described protocols [55,56]. Briefly, immortalized mouse subcutaneous connective tissue fibroblasts (NCTC clone 929 [L-cell, L-929, Strain L derivative]; American Type Culture Collection (ATCC), Manassas, VA, USA) (CCL1) or HGF (Applied Biological Materials, Inc., Richmond, BC, Canada) were exposed to 2-fold serial dilutions (AM_sil1_: ≤8.34 mmol/L; AM_sil2_: ≤7.28 mmol/L). Chosen concentrations corresponded to approx. 7% mass fraction of AM_sil1_ or AM_sil2_ in the copolymer resin and a maximum of 2% leaching. To allow for the possibility of restoration multiplicity and variable size, a 2-fold greater dilution was also included in the testing. These calculations are based on the accelerated leachability study of UDMA/PEG-U/2-hydroxyethyl methacrylate (HEMA) resin (abbreviated UPH; a close analog to UPE resin used in this study) and ACP-UPH composites [57]. After 24 and 72 h incubation, cells were assessed for cell viability (LIVE/DEAD^®^ Viability/Cytotoxicity kit, Life Technologies, Corp., Grand Island, NY, USA) and metabolic activity (CellTiter^®^ AQueous One Solution Reagent; Promega, Corp., Madison, WI, USA). Controls were without the AM_sil_s and/or cells. The CCL1 cells and HGFs were maintained, at 37 °C and 5% CO_2_, in 10% serum-supplemented Eagle’s minimum essential medium (ATCC) and PriGrow III medium (Applied Biological Materials, Inc.), respectively. For experiments, cells were obtained from a subconfluent stock culture. Means were obtained from 5 independent replicates tested in duplicate.

### 4.5. Contact Angle (CA)

Changes in hydrophilicity/hydrophobicity of UPE resins due to the introduction of AM_sil_s were assessed by CA measurements (drop shape analyzer DSA100, Krüss GmbH, Hamburg, Germany). Following the deposition of the sessile droplets of the resin on the substrate, they were imaged after 1 min resting time with a charge-coupled device camera at the points of intersection (three-phase contact points) between the drop contour and the projection of the surface (baseline). The CA water values were calculated employing the Krüss Advance software. Four repetitive measurements were performed in each group.

### 4.6. Copolymer Specimen Preparation

For biotesting, UPE and AM_sil1_–UPE and AMsi_l2_–UPE copolymer specimens were fabricated by filling circular openings of a flat stainless-steel molds (6 mm diameter, 0.5 mm thickness) with the resins. Each side of the mold was covered with Mylar film and a glass slide, firmly clamped, and then cured (2 min/side: Triad 2000; Dentsply International, York, PA, USA).

Specimens were subjected to extraction in Dulbecco’s phosphate-buffered saline lacking both calcium and magnesium (Life Technologies, Grand Island, NY, USA) for 72 h, at 37 °C and 6.3 rad/s using an orbital shaker. After extraction, the disks were dried under vacuum (desiccator; ~22 °C) for 7 days. Specimens were sterilized for 12 h using an Anprolene gas sterilization chamber (Andersen Products, Inc., Haw River, NC, USA). Prior to bacterial testing, specimens were degassed for ≥5 days under vacuum (desiccator; ~22 °C).

### 4.7. Degree of Vinyl Conversion (DVC)

DVC of UPE and (AM_sil1_ or AM_sil2_)–UPE resins was determined by collecting the near-IR (NIR) spectra (Nexus; ThermoFisher, Madison, WI, USA) before and 24 h after the light cure and calculating the reduction in =C–H absorption band at 6165 cm^−1^ in the overtone region in going from monomers to polymers. By maintaining a constant specimen thickness, a need for an invariant internal standard was eliminated. The DVC was calculated as

DVC (%) = [(area_monomer_ − area_polymer_)/area_monomer_] **×** 100 (1)

where area_polymer_ and area_monomer_ correspond to the areas under 6165^−1^ absorption peak after and before the polymerization, respectively.

### 4.8. Mechanical Properties of Copolymers

Test specimens (2 mm × 2 mm × 25 mm) for flexural strength (FS) and elastic modulus (E) determinations were photopolymerized in the same manner as the copolymer disks for biological testing. Polymerized specimens did not undergo any additional treatment. The FS and E of UPE and (AM_sil1_ or AM_sil2_) UPE copolymer specimens was tested employing the Universal Testing Machine (Instron 5500R, Instron Corp., Canton, MA, USA). The load was applied (crosshead speed of 1 mm/min) to the center of a specimen positioned on a test device with supports 20 mm apart. The FS and E of the specimens (three replicates/experimental group) were calculated as instructed in the ISO4049:2009 document.

### 4.9. Bacterial Testing

#### 4.9.1. Planktonic

Testing of *Streptococcus mutans* UA-159 (ATCC^®^ 700610) planktonic forms was according to described methods [47,58]. Briefly, bacterial cultures with an optical density of 1.2–1.3 at 600 nm (Unico^®^ 1200 Spectrophotometer, United Products & Instruments, Inc., Dayton, NJ, USA) were diluted and seeded onto copolymer disks at a density of ~3 × 10^7^ CFU/disk. Another disk was placed atop to maximize contact. After a 2 h incubation (37 °C, 5% CO_2_) the samples were placed in 1 mL of Todd Hewitt broth, rigorously mixed, and utilized to prepare a 10-fold dilution series. A 100 µL aliquot of the resulting suspensions were spread onto the surface of THB agar plates. After incubation (~20 h at 37 °C, 5% CO_2_), colony-forming units were enumerated using an IncuCount Colony Counter (Revolutionary Science, Shafer, MN, USA). UPE resin disks and HemCon^®^ Dental Dressing (HemCon Medical Technologies, Inc., Portland, OR, USA) were used as negative and positive controls, respectively. Around 30 to 300 CFU per spread plate was the range considered countable. Notwithstanding, agar plates streaked with neat solutions of some groups yielded less than the lower limit of detection. Such data were reported as less than the limit of quantification. The number of CFU/mL was calculated as CFU number/(volume plated × dilution factor). Mean counts were obtained from five independently tested copolymer disk sandwiches.

#### 4.9.2. Biofilm

A bioluminescent *S. mutans* strain JM 10 (derivative of wild type UA159 [59] was used to assess the AM properties of AM_sil_s_._ Methods of the real-time bioluminescence assay were as described [41,58].

*Porphyromonas gingivalis*, strain FDC 381 (ATCC^®^ BAA-1703) was propagated in Becton Dickinson BBL chopped meat carbohydrate, pre-reduced II broth, using a shaking incubator (37 °C, anaerobic conditions). Three-day cultures were diluted in broth to approximate 5 × 10^6^ CFU/mL. Copolymer disks, vertically supported in a 24-well plate, were immersed in 1.6 mL of the bacterial suspension. In anaerobic conditions, the plate was incubated at 37 °C for 4 days. The copolymer disks were washed thrice in sterile 0.89% NaCl solution. Thereafter, the biofilm was displaced from the copolymer disks by transferring them to a sterile glass tube containing 1 mL of saline, vortexed (1 min), sonicated (10 min), and vortexed (1 min). Each disk was visually examined to ensure that the biomass was removed. The resulting suspensions were used to make 10-fold serial dilutions and subsequently spread onto the surface of Brucella agar with hemin and vitamin K1 (Sigma-Aldrich, St. Louis, MO, USA) plates. After incubation (3 days at 37 °C, anaerobic conditions), colony-forming units were enumerated.

### 4.10. Statistical Analyses

Analysis of variance and multiple paired comparisons (two-sided, 95% confidence interval) were used to analyze the experimental data as a function of material makeup and/or exposure/incubation time and establish a statistical significance of differences between the experimental groups. Correlation coefficient (*r*) was calculated to determine the functional dependence between cellular metabolic activity and viability. (SigmaPlot™, Systat Software, San Jose, CA, USA and/or Microsoft Office Excel 2016; Microsoft, Redmond, WA, USA). Graphics were created using Microsoft Office Excel 2016 and/or DeltaGraph6 for Windows^®^ (Red Rock Software, Inc., Salt Lake City, UT, USA).

## Figures and Tables

**Table 1:** Assignments of ^13^C and ^1^H NMR chemical shifts of AM_sil1_.

Atom #	^13^C Chemical Shift, ppm	^1^H Chemical Shift, ppm	# of H’s	Signal Splitting
1	126.6 (CH_2_)	5.77, 6.09	1, 1	singlets
2	135.3 (C)		0	
3	17.8 (CH_3_)	1.92	3	singlet
4	165.8 (C)		0	
5	58.0 (CH_2_)	4.52	2	multiplet
6	61.7 (CH_2_)	3.70	2	multiplet
7, 8	50.6 (CH_3_)	3.09	6	singlet
9	65.9 (CH_2_)	3.34	2	multiplet
10	15.6 (CH_2_)	1.72	2	multiplet
11	5.2 (CH_2_)	0.54	2	multiplet
12, 13, 14	50.1 (CH_3_)	3.51	9	singlet

Atom numbering is illustrated in Figure 1.

**Table 2:** Assignments of ^13^C and ^1^H NMR chemical shifts of AM_sil2_.

Atom #	^13^C Chemical Shift, ppm	^1^H Chemical Shift, ppm	# of H’s	Signal Splitting
1	126.5 (CH_2_)	5.76, 6.08	1, 1	singlets
2	135.3 (C)		0	
3	17.8 (CH_3_)	1.91	3	singlet
4	165.8 (C)		0	
5	58.1 (CH_2_)	4.52	2	multiplet
6	61.6 (CH_2_)	3.70	2	multiplet
7, 8	50.4 (CH_3_)	3.09	6	singlet
9	63.8 (CH_2_)	3.36	2	multiplet
10	21.7 (CH_2_)	1.67	2	multiplet
11–18	22.1, 25.7, 28.4, 28.6, 28.7, 28.8, 28.9, 32.3 (CH_2_)	1.25	16	multiplet
19	8.6 (CH_2_)	0.57	2	multiplet
20, 21, 22	49.9 (CH_3_)	3.46	9	singlet

Atom numbering is illustrated in Figure 1.

## Abbreviations

ACP	amorphous calcium phosphate
AM	antimicrobial
AMRE	antimicrobial and remineralizing
AM_sil1_	N-(2-(methacryloyloxy)ethyl-*N*,*N*-dimethyl-3-(trimethoxysilyl)propan-1-aminium iodide
AM_sil2_	N-(2-(methacryloyloxy)ethyl-*N*,*N*-dimethyl-3-(trimethoxysilyl)undecan-1-aminium bromide
Bis-GMA	2,2-bis[p-(2-hydroxy-3-methacryloxypropoxy)phenyl]propane
BrUDTMS	(11-bromoundecyl)trimethoxy silane
CA	contact angle
CCL1	immortalized mouse subcutaneous connective tissue fibroblasts
CQ	camphorquinone
DMAEMA	2-(dimethylamino)ethyl methacrylate
DVC	degree of vinyl conversion
4EDMAB	ethyl-4-*N*,*N*-dimethylamino benzoate
EHMA	ethyl 2-(hydroxymethyl) acrylate
HGF	human gingival fibroblasts
IPTMS	(3-iodopropyl)trimethoxy silane
MDBP	methacryloyloxydodecyl pyrimidinium bromide
NMR	nuclear magnetic resonance
PEG-U	poly(ethylene glycol)-extended UDMA
QA	quaternary ammonium
TEGDMA	triethyleneglycol dimethacrylate
UDMA	urethane dimethacrylate
UPE	UDMA/PEG-U/EHMA resin

## References

[1] Gluzman R., Katz R.V., Frey B.J., McGowan R. (2013). Prevention of root caries: A literature review of primary and secondary preventive agents. Spec. Care Dentist..

[2] Cramer N.B., Stansbury J.W., Bowman C.N. (2011). Recent advances and developments in composite dental restorative materials. J. Dent. Res..

[3] Ferracane J.L. (2011). Resin composite—state of the art. Dent. Mater..

[4] Moreau J.L., Xu H.H. (2010). Fluoride releasing restorative materials: Effects of pH on mechanical properties and ion release. Dent. Mater..

[5] Wiegand A., Buchalla W., Attin T. (2007). Review on fluoride-releasing restorative materials--fluoride release and uptake characteristics, antibacterial activity and influence on caries formation. Dent. Mater..

[6] Imazato S. (2009). Bio-active restorative materials with antibacterial effects: New dimension of innovation in restorative dentistry. Dent. Mater. J..

[7] Pereira-Cenci T., Cenci M.S., Fedorowicz Z., Marchesan M.A. (2009). Antibacterial agents in composite restorations for the prevention of dental caries. Cochrane Database Syst. Rev..

[8] Dallas P., Sharma V.K., Zboril R. (2011). Silver polymeric nanocomposites as advanced antimicrobial agents: Classification, synthetic paths, applications, and perspectives. Adv. Colloid Interface Sci..

[9] Jedrychowski J.R., Caputo A.A., Kerper S. (1983). Antibacterial and mechanical properties of restorative materials combined with chlorhexidines. J. Oral Rehabil..

[10] Kawahara K., Tsuruda K., Morishita M., Uchida M. (2000). Antibacterial effect of silver-zeolite on oral bacteria under anaerobic conditions. Dent. Mater..

[11] Knetsch M.L., Koole L.H. (2011). New strategies in the development of antimicrobial coatings: The example of increasing usage of silver and silver nanoparticles. Polymers.

[12] Osinaga P.W., Grande R.H., Ballester R.Y., Simionato M.R., Delgado Rodrigues C.R., Muench A. (2003). Zinc sulfate addition to glass-ionomer-based cements: Influence on physical and antibacterial properties, zinc and fluoride release. Dent. Mater..

[13] Syafiuddin T., Hisamitsu H., Toko T., Igarashi T., Goto N., Fujishima A., Miyazaki T. (1997). In vitro inhibition of caries around a resin composite restoration containing antibacterial filler. Biomaterials.

[14] Takahashi Y., Imazato S., Kaneshiro A.V., Ebisu S., Frencken J.E., Tay F.R. (2006). Antibacterial effects and physical properties of glass-ionomer cements containing chlorhexidine for the ART approach. Dent. Mater..

[15] Yoshida K., Tanagawa M., Atsuta M. (1999). Characterization and inhibitory effect of antibacterial dental resin composites incorporating silver-supported materials. J. Biomed. Mater. Res..

[16] Weng Y., Guo X., Chong V.J., Howard L., Gregory R.L., Xie D. (2011). Synthesis and evaluation of a novel antibacterial dental resin composite with quaternary ammonium salts. J. Biomed. Sci. Eng..

[17] Makvandi P., Jamaledin R., Jabbari M., Nikfarjam N., Borzacchiello A. (2018). Antibacterial quaternary ammonium compounds in dental materials: A systematic review. Dent. Mater..

[18] Xue Y., Xiao H., Zhang Y. (2015). Antimicrobial polymeric materials with quaternary ammonium and phosphonium salts. Int. J. Mol. Sci..

[19] Gottenbos B., van der Mei H.C., Klatter F., Nieuwenhuis P., Busscher H.J. (2002). In vitro and in vivo antimicrobial activity of covalently coupled quaternary ammonium silane coatings on silicone rubber. Biomaterials.

[20] Lee S.B., Koepsel R.R., Morley S.W., Matyjaszewski K., Sun Y., Russell A.J. (2004). Permanent, nonleaching antibacterial surfaces. 1. Synthesis by atom transfer radical polymerization. Biomacromolecules.

[21] Lu G., Wu D., Fu R. (2007). Studies on the synthesis and antibacterial activities of polymeric quaternary ammonium salts from dimethylaminoethyl methacrylate. React. Funct. Polym..

[22] Li F., Chen J., Chai Z., Zhang L., Xiao Y., Fang M., Ma S. (2009). Effects of a dental adhesive incorporating antibacterial monomer on the growth, adherence and membrane integrity of Streptococcus mutans. J. Dent..

[23] Thome T., Mayer M.P., Imazato S., Geraldo-Martins V.R., Marques M.M. (2009). In vitro analysis of inhibitory effects of the antibacterial monomer MDPB-containing restorations on the progression of secondary root caries. J. Dent..

[24] Antonucci J.M. (2012). Polymerizable biomedical composition. U.S. Patent.

[25] Antonucci J.M., Zeiger D.N., Tang K., Lin-Gibson S., Fowler B.O., Lin N.J. (2012). Synthesis and characterization of dimethacrylates containing quaternary ammonium functionalities for dental applications. Dent. Mater..

[26] Li F., Chai Z.G., Sun M.N., Wang F., Ma S., Zhang L., Fang M., Chen J.H. (2009). Anti-biofilm effect of dental adhesive with cationic monomer. J. Dent. Res..

[27] Boskey A.L. (1997). Amorphous calcium phosphate: The contention of bone. J. Dent. Res..

[28] Dorozhkin S.V. (2011). Biocomposites and hybrid biomaterials based on calcium orthophosphates. Biomatter.

[29] Dorozhkin S.V. (2011). Calcium orthophosphates: Occurrence, properties, biomineralization, pathological calcification and biomimetic applications. Biomatter.

[30] He G., Dahl T., Veis A., George A. (2003). Nucleation of apatite crystals in vitro by self-assembled dentin matrix protein 1. Nat. Mater..

[31] Tsuji T., Onuma K., Yamamoto A., Iijima M., Shiba K. (2008). Direct transformation from amorphous to crystalline calcium phosphate facilitated by motif-programmed artificial proteins. Proc. Natl. Acad. Sci. USA.

[32] Weiner S. (2006). Transient precursor strategy in mineral formation of bone. Bone.

[33] Weiner S., Sagi I., Addadi L. (2005). Structural biology. Choosing the crystallization path less traveled. Science.

[34] Tadic D., Peters F., Epple M. (2002). Continuous synthesis of amorphous carbonated apatites. Biomaterials.

[35] Antonucci J.M., Skrtic D. (2010). Fine-tuning of polymeric resins and their interfaces with amorphous calcium phosphate. A strategy for designing effective remineralizing dental composites. Polymers.

[36] Skrtic D., Antonucci J.M. (2007). Dental composites based on amorphous calcium phosphate - resin composition/physicochemical properties study. J. Biomater. Appl..

[37] Skrtic D., Antonucci J.M., Eanes E.D. (2003). Amorphous calcium phosphate-based bioactive polymeric composites for mineralized tissue regeneration. J. Res. Natl. Inst. Stan..

[38] Skrtic D., Antonucci J.M., Eanes E.D., Eidelman N. (2004). Dental composites based on hybrid and surface-modified amorphous calcium phosphates. Biomaterials.

[39] Zhang F., Allen A.J., Levine L.E., Vaudin M.D., Skrtic D., Antonucci J.M., Hoffman K.M., Giuseppetti A.A., Ilavsky J. (2014). Structural and dynamical studies of acid-mediated conversion in amorphous-calcium-phosphate based dental composites. Dent. Mater..

[40] Okeke U.C., Synder C.R., Frukhtbeyn S.A. (2019). Synthesis, purification and characterization of polymerizable multifunctional quaternary ammonium compounds. Molecules.

[41] Esteban Florez F.L., Hiers R.D., Smart K., Kreth J., Qi F., Merritt J., Khajotia S.S. (2016). Real-time assessment of Streptococcus mutans biofilm metabolism on resin composite. Dent. Mater..

[42] Forssten S.D., Bjorklund M., Ouwehand A.C. (2010). Streptococcus mutans, caries and simulation models. Nutrients.

[43] Fenesy K.E. (1998). Periodontal disease: An overview for physicians. Mt. Sinai J. Med..

[44] Rafiei M., Kiani F., Sayehmiri K., Sayehmiri F., Tavirani M., Dousti M., Sheikhi A. (2018). Prevalence of Anaerobic Bacteria (P.gingivalis) as Major Microbial Agent in the Incidence Periodontal Diseases by Meta-analysis. J. Dent..

[45] Antonucci J.M., Davis C.H., Sun J., O’Donnell J.N., Skrtic D. (2011). Leachability and Cytotoxicity of an Experimental Polymeric ACP Composite. PMSE Prepr. Am. Chem. Soc. Div. Polym. Mater. Sci. Eng. Meet..

[46] Da Silva E.M., Almeida G.S., Poskus L.T., Guimaraes J.G. (2008). Relationship between the degree of conversion, solubility and salivary sorption of a hybrid and a nanofilled resin composite. J. Appl. Oral Sci..

[47] Bienek D.R., Giuseppetti A.A., Skrtic D., Dutour-Sikiric M., Furedi-Milhofer H. (2019). Amorphous calcium phosphates as bioactive filler in polymeric dental composites. Calcium Phosphates-From Fundamentals to Applications.

[48] Sideridou I., Tserki V., Papanastasiou G. (2002). Effect of chemical structure on degree of conversion in light-cured dimethacrylate-based dental resins. Biomaterials.

[49] Silhavy T.J., Kahne D., Walker S. (2010). The bacterial cell envelope. Cold Spring Harb. Perspect. Biol..

[50] Chen C.Z., Beck-Tan N.C., Dhurjati P., van Dyk T.K., LaRossa R.A., Cooper S.L. (2000). Quaternary ammonium functionalized poly (propylene imine) dendrimers as effective antimicrobials: Structure−activity studies. Biomacromolecules.

[51] Ikeda T., Hirayama H., Yamaguchi H., Tazuke S., Watanabe M. (1986). Polycationic biocides with pendant active groups: Molecular weight dependence of antibacterial activity. Antimicrob. Agents Chemother..

[52] Ikeda T., Yamaguchi H., Tazuke S. (1990). Molecular weight dependence of antibacterial activity in cationic disinfectants. J. Bioact. Compat. Polym..

[53] Kapralos V., Koutroulis A., Ørstavik D., Sunde P.T., Rukke H.V. (2018). Antibacterial Activity of Endodontic Sealers against Planktonic Bacteria and Bacteria in Biofilms. J. Endod..

[54] Harrison J.J., Ceri H., Stremick C., Turner R.J. (2004). Differences in biofilm and planktonic cell mediated reduction of metalloid oxyanions. FEMS Microbiol. Lett..

[55] Bienek D.R., Frukhtbeyn S.A., Giuseppetti A.A., Okeke U.C., Pires R.M., Antonucci J.M., Skrtic D. (2018). Ionic dimethacrylates for antimicrobial and remineralizing dental composites. Ann. Dent. Oral Disord..

[56] Bienek D.R., Frukhtbeyn S.A., Giuseppetti A.A., Okeke U.C., Skrtic D. (2018). Antimicrobial monomers for polymeric dental restoratives: Cytotoxicity and physicochemical properties. J. Funct. Biomat..

[57] Skrtic D., Antonucci J.M. (2011). Bioactive polymeric composites for tooth mineral regeneration: Physicochemical and cellular aspects. J. Funct. Biomat..

[58] Bienek D.R., Giuseppetti A.A., Okeke U.C., Frukhtbeyn S.A., Dupree P.J., Khajotia S.S., Esteban Florez F.L., Hiers R.D., Skrtic D. (2019). Antimicrobial, biocompatibility, and physicochemical properties of novel adhesive methacrylate dental monomers. J. Bioact. Compat. Polym..

[59] Merritt J., Kreth J., Qi F., Sullivan R., Shi W. (2005). Non-disruptive, real-time analyses of the metabolic status and viability of Streptococcus mutans cells in response to antimicrobial treatments. J. Microbiol. Methods.

